# External costs of electricity generation in 27 European countries from 2010–2030: Pathway toward sustainability or business as usual?

**DOI:** 10.1371/journal.pone.0294499

**Published:** 2024-02-23

**Authors:** Frank Baumgärtner, Peter Letmathe

**Affiliations:** School of Business and Economics, RWTH Aachen University, Aachen, Germany; Alexandru Ioan Cuza University: Universitatea Alexandru Ioan Cuza, ROMANIA

## Abstract

Electricity generation in Europe is undergoing a fundamental change. The aim is to increase sustainability by reducing emissions. Each country has a different electricity mix, and there is no established method for measuring environmental impacts of electricity production with a single monetary indicator, in a uniform manner, and with country-specific data. To address this gap, a model that measures the costs of 19 environmental externalities (usually, types of emissions) has been developed. Using country-specific technologies, electricity mixes, and external cost rates, the development of external costs of generating electricity in 27 European countries between 2010 and 2030 is assessed and analyzed. The simulation results show that the external costs vary heavily between 2.1 and 22.4 euro cents per kWh in this period. Despite the initiated transformation of the energy systems in many EU countries, external costs per kWh are decreasing in only eight of them. This fact underlines the need for a drastic change in national energy strategies. Overall, the results show that more far-reaching policy measures are needed in order to significantly reduce the external costs of the energy sector in Europe. The article raises the level of granularity of research on the external costs of electricity in Europe by combining extensive country-specific emission data and country-specific external cost rates.

## Introduction

The energy sector in Europe is undergoing a profound transformation process [1] for which the European Union (EU) has set ambitious goals. In December 2019, the European Commission (EC) announced its "Green Deal". Building upon its earlier decisions, the EC now plans to regularly adjust its original goals and to become climate-neutral by 2050 [2]. To fulfill the Paris Agreement, it plans to invest at least one trillion € in the electricity infrastructure [3]. Most recently, the classification of gas-fired and nuclear power plants as "green" technologies [4] according to the EU taxonomy for sustainable activities [5] has caused discussion in this context. The electricity sector is of particular relevance to sustainability considerations, as it is not only the sector with the highest greenhouse gas (GHG) emissions [6] but also with the largest impact on human health [7,8]. The measures and pathways for achieving these sustainability goals have not yet been specified. Thus, the governments of the respective EU member states are free to determine their own transformation pathways [9–15]. To quantitatively and aggregately assess the environmental dimensions of sustainability, external costs in particular are appropriate for illustrating negative consequences caused by the energy sector [16,17] which are not borne by producers or consumers. With the notable exception of carbon dioxide (CO_2_) [18], these costs are usually not taken into account in business decisions even though they are of vital importance [19–21].

The main objective of this paper is to provide a comprehensive approach to the assessment of external costs of electricity for further research. The second objective is to identify the external costs of electricity in Europe for the general public, and in particular for policy makers, in order to provide recommendations for an environmentally sound development. Overall, this article shows how the external costs of the individual countries of the European Union are likely to develop from 2010 to 2030, using comprehensive national emissions data and national external cost rates for relevant environmental externalities. In total, 19 different environmental externalities are considered for all technologies.

While most environmental assessments are evaluated using impact categories [22,23], evaluation with external cost rates offers significant advantages for general comprehensibility [24,25] as well as for decision makers, since external costs can be directly related to monetary investments or costs der [26,27]. Most studies on external costs of electricity examine the system only in one country or region [28–34]. Studies that compare the externality costs of electricity in different countries usually focus on a small number of externalities [20,21,35–43]. Generally, these studies face the challenge of combining country-specific technology data with country-specific external cost rates. To the authors’ knowledge, no study comparing different countries has a similar level of detail of country-specific data, so the following study is particularly suited to determine the environmental damage of electricity production and to provide a basis for policy recommendations for changes in European electricity systems. Thus, the article goes significantly beyond the previous state of research, as will be demonstrated in more detail in the following chapter.

## Materials and methods

### Literature review

To assess the environmental impacts of electricity production, lifecycle assessments are usually applied in the literature [22,23]. A lifecycle assessment first consists of the definition of goal and scope, then an inventory analysis is carried out and the results of it are evaluated in the impact assessment by impact categories and finally interpreted [44]. However, there is no uniform framework according to which studies are conducted. Different impact assessment methods are used, and the selected impact categories also vary greatly between studies [22,23]. Extensive ecological results can be obtained through different impact categories, but the results are difficult to understand for the general public and thus the application of lifecycle assessment for decision-making is also limited [45]. In addition to the formation of endpoint indicators, it would be possible to aggregate the results into efficiency scores using data envelopment analysis [46]. However, one of the main challenges is the weighting of the impact categories. These are generally calculated in the model, whereas an alternative would be to define them explicitly, for instance based on expert interviews [47].

In comparison to impact categories, external costs as an alternative form of impact assessment are more suitable for policy makers. By aggregating the results into a monetary measure, for example, the costs and benefits of interventions can be contrasted. In the same way, market interventions such as taxes or subsidies can be evaluated for their effectiveness in reducing costs otherwise borne by the society [27,48,49]. Moreover, external costs are an effective tool for comparing technologies or externalities across countries [50].

Numerous research works evaluate the external costs of electricity production within a single country or for a single technology, but only a few studies compare the external costs of electricity production between different countries [20,21,35–43]. These latter studies face the extra challenge that a mere aggregation of power plant technology-specific emissions into a country-specific energy mix is inadequate, as even the emissions from the same technologies differ across countries [7,51]. This fact results from technical differences, e.g., due to different efficiency levels or emissions abatement technologies [52] but also differences in other non-technical factors (like fuel inputs or operational practices). Furthermore, country-specific cost rates are relevant for multiple air pollutants in order to account for the background concentration of the pollutant and how heavily an area is inhabited [53,54].

In addition, there is no established framework regarding which emissions should be included in external cost calculations. Many studies focus exclusively on GHGs [55,56]. Nevertheless, ambient air pollution is a crucial driver of human health impacts [51,57]. However, air pollutants are usually only fundamentally considered in terms of sulfur oxide (SO_2_), nitrogen oxides (NO_X_), and particulates (PM) [7,20,51,58–62]. No previous study adequately addresses these issues.

In this sense, this study advances the state of the art by being the first to determine the external costs of electricity generation in 27 European countries at a high level of detail. At the inventory analysis level, country-specific technologies and their environmental externalities are included. By means of energy scenarios, the emissions of the average electricity mix are determined for the period between 2010 and 2030 in the different countries. In addition, country-specific and year-specific external cost rates are utilized for the impact assessment. In this way, the present study surpasses previous research approaches.

In this article, the following 27 EU countries in 2010, the start date of the analysis, are considered (referred to as EU countries): Austria (AT), Belgium (BE), Bulgaria (BG), Cyprus (CY), the Czech Republic (CZ), Germany (DE), Denmark (DK), Estonia (EE), Spain (ES), Finland (FI), France (FR), Greece (GR), Hungary (HU), Ireland (IE), Italy (IT), Lithuania (LT), Luxembourg (LU), Latvia (LV), Malta (MT), the Netherlands (NL), Poland (PL), Portugal (PT), Romania (RO), Sweden (SE), Slovenia (SI), Slovakia (SK) and the United Kingdom (UK). These are the 27 members of the EU before the accession of Croatia in 2013 and the Brexit (i.e. the UK’s withdrawal from the EU). The selection is based on the availability of technological data. To simplify, the article nevertheless refers to these 27 countries as both European countries and EU member states in the article, since the results of the analysis of the 27 European countries have great significance for the results of the actual EU today due to the large overlap. For each country k, the Electricity Mix Matrix B^k^, the Technology Matrix A^k^ and the External Costs Rates Matrix C^k^ are determined. In the following, the methodical procedure for deriving these matrices and for collecting the data used is described.

### Electricity mix matrix B^k^

To determine the Electricity Mix Matrix B^k^ for each of the 27 countries under consideration, the electricity mixes at technology level in 2010, 2020 and 2030 for the corresponding country were obtained from the eco-database ProBas+ [63], which were accessed via the open-source and free software for sustainability and life cycle assessment openLCA 1.7.4 [64]. The technologies used and their percentage composition of the respective electricity mixes were determined from the unit processes (UP) of a country’s power plant parks in the corresponding years.

Linear changes in the market share over the two decades are assumed to determine the market shares of all technologies for each year. However, for the sake of clarity, these interim values are not considered further in this article. The energy mixes for the individual years were plotted column by column in a table for each country.

With the procedure described, the Electricity Mix Matrix B^k^ is formed, which is shown in Eq (1). The matrix B^k^ consists of 21 columns for each country, representing the individual electricity mix in the years from 2010 to 2030. The electricity mix is composed of the technologies arranged in the rows.


$$\begin{array}{c} B^{k}=\left(\begin{array}{ccc} b_{11}^{k} & \cdots & b_{1|L|}^{k}\\ \vdots & \ddots & \vdots \\ b_{|J|1}^{k} & \cdots & b_{\left|J||L\right|}^{k} \end{array}\right)=(b_{jl}^{k})\\ B^{k}\in R^{\left|J|\times |L\right|},j\in J=\{Technologies\},l\in L=\{Years\}=\{2010,\ldots ,2030\},k\in K=\{EU\;countries\},|L|=21 \end{array}$$ (1)

### Technology matrix A^k^

Similar to the Electricity Mix Matrix B^k^, the emissions of all the technologies that generate electricity were obtained, in order to set up the Technology Matrix for each country. First, the Life Cycle Inventories (LCI), also called System Processes, of all electricity technologies were exported from the ProBas+ database [63] analogously to the UPs of the power plant parks. The number of technologies is extensive, since for energy conversion technologies there is a distinction depending on the location of the plant and, in some cases, on the technology level.

The LCIs include the individual emissions of the technologies, taking into account the direct emissions from the power plants and emissions in the upstream supply chain. For all technologies, in addition to the GHG emissions, CO_2_, methane (CH_4_), and nitrous oxide (N_2_O), data are also available for emissions of air pollutants often considered in other studies, such as NO_X_, PM, and SO_2_. Furthermore, the emissions of ammonia (NH_3_), arsenic (As), cadmium (Cd), carbon monoxide (CO), chromium (Cr), dioxins, lead (Pb), mercury (Hg), nickel (Ni), and non-methane volatile organic compounds (NMVOCs) are included. Since no specific subdivision of the PMs is available, it is assumed that half of the mass is attributable to the PM_2.5_ class (diameter of 2.5 μm or less) and the other half to the PM_coarse_ class (diameter between 2.5 and 10 μm). There are partial data that the distribution differs between electricity generation options [65,66], but there are no reliable values for all technologies. However, a corresponding adjustment would change the results only marginally. The reference unit for all emissions is the emission per terajoule (TJ) of electricity generated in a power plant. The system boundary of the ProBas+ data covers the entire material flows from cradle to gate. In accordance with the authors, a uniform surcharge of 5% for transmission and distribution losses to the final electricity consumer is assumed [63]. The emission values are representative of typical emissions of the respective conversion technology to generate one TJ of electricity in the respective country and not of individual power plant measurements. Emissions to water and soil were neglected. Besides the emissions of pollutants, specific externalities of nuclear power plants, such as radioactive radiation during regular operation, disposal of fuel elements, and the risk of a nuclear accident, are included in the model. In the Technology Matrix A^k^, these are only stored as dummy variables (1 for nuclear power plants, 0 for all other technologies). A specific evaluation is performed based on the literature within the External Costs Rates Matrix C^k^.

A particularly noteworthy aspect of the approach is the possibility of combining electricity mixes based on country-specific technologies with the specific emissions of individual technologies. Among other things, the emissions of individual power generation technologies differ over time and between countries. The technologies that constitute the electricity mixes in Matrix B^k^ are therefore listed in the same sequence in Technology Matrix A^k^. However, the technologies here are located in the rows of the matrix A^k^, while the 19 environmental externalities considered are listed in the columns. Eq (2) illustrates this.


$$\begin{array}{c} A^{k}=\left(\begin{array}{ccc} a_{11}^{k} & \cdots & a_{1|J|}^{k}\\ \vdots & \ddots & \vdots \\ a_{|I|1}^{k} & \cdots & a_{\left|I||J\right|}^{k} \end{array}\right)=(a_{ij}^{k})\\ A^{k}\in R^{\left|I|\times |J\right|},i\in I=\{Externalities\},j\in J=\{Technologies\},\;k\in K=\{EU\;countries\},|I|=19 \end{array}$$ (2)

Data from the ProBas+ database [63] are applied to determine the technologies used to produce electricity in European countries, their emissions, and the technologies leading to the respective electricity mixes. Other sources also contain average emissions of electricity generation technologies or electricity mixes for countries [67,68]. However, there is a lack of country-specific data on emissions from individual technologies used in countries. Based on this, there should also be detailed electricity mixes and outlooks for these. ProBas+ divides the electricity mixes more comprehensively than the other sources into sub-technologies and allows changing emissions over time for technologies. However, ProBas+ only offers one trajectory for its development up to 2030.

Different scenarios would be desirable in this regard. If comprehensive scenarios do become available, the ecological consequences could be evaluated with the methodology described in the article. In the same vein, an application to other regions is a promising research avenue if data are available. In this respect, the idea of expressing environmental impacts as external costs can be very suitable to economically value improvements in the energy sector. In such a way, impacts can also be more accessible to a general audience than more specific indicators are.

### External costs rates matrix C^k^

External cost rates are the monetized impact assessment of an environmental externality per unit. This article uses the extensive database of the European NEEDS (New Energy Externalities Developments for Sustainability) project, which is part of the Research and Technological Development Framework Programme of the European Union as the primary source for the assessment of air pollutants. In the project, external cost rates were determined for European countries in Research Stream 3a [69], taking into account, for example, the average background concentrations and the number of people affected. The approach considers damage to human health in terms of mortality and morbidity. Since human life is fundamentally irreplaceable, willingness-to-pay or willingness-to-accept approaches were used. Mortality, for example, results in damages of 3 million € [70]. In addition, the approach also takes the costs of crop losses and biodiversity losses into account [69].

It is possible to normalize the costs to the monetary value in one year. The year 2019 was set as the index year. In accordance with the accompanying document of the database[69], an income elasticity of 0.85, a time preference rate of 1% and an average rate of growth of the gross domestic product (GDP) up to 2030 of 2% are assumed, in order to obtain the actual cost rates for each individual year of the study from 2010 to 2030. Further information is provided in the corresponding manual by Friedrich and Bickel on external costs and the approach of the NEEDS project [48].

The missing cost rate for carbon monoxide (CO) in the NEEDS project is determined uniformly for all countries in the analysis, based on a literature review by Matthews and Lave [71]. The provided median external cost rate of the reviewed studies is chosen, taking into account the relevant exchange rate and the purchasing power parity (PPP) according to the guidelines of the NEEDS project [69]. Further adjustments within the assessment period were made in the same way as for the other pollutants.

As stated, the article relies on the NEEDS database [69]. Although more other European databases to assess air pollutants are now available [27,72,73], they do not have the same extent. In addition, they are all based on the NEEDS database and the NEEDS methodology also provides adjustments of external cost rates for different years.

In the EU study [27], the possibility of evaluating emissions, such as various heavy metals, with their external cost rate has been discontinued. In addition, it is no longer possible to distinguish between different levels of emissions, and there is only one aggregated cost rate for each emission. A subdivision into different causes (e.g., loss of biodiversity or human health) is no longer made. The Manual of External Costs of the CE Delft [72] also does not offer the possibilities of subdividing the cost rates and the different evaluations according to emission levels. Although there is an evaluation of the various pollutants such as heavy metals, only a country-specific cost rate for the Netherlands and an average European value are available for all emissions. The latest study by the German Federal Environment Agency [73] again includes different evaluations according to emission levels and allows for the subdivision of the cost rates according to the different causes of emissions. Still, the pricing of heavy metals is missing here. In addition, the study reports external cost rates for Germany only.

In summary, it can be stated that these studies generally arrive at quite similar estimates for the external cost rates due to their mutual reference to the NEEDS database. Furthermore, sensitivity test indicate no relevant changes in the results. Some studies assume even higher health effects, especially from PM_2.5_ [74], but so far there has been no clear assessment of these impacts concerning the external cost rates of air pollutants in Europe. In lifecycle assessments, there are also approaches for a spatially high-resolution assessment of emissions [75,76]. Here, it would be interesting to find out how they would affect external cost rates. This study, however, only evaluates the external costs of an average unit of electricity, which is why this topic does not affect further in the article.

Although the damage caused by GHGs does not differ with regard to the country where GHGs are emitted, the monetary damage is dependent on the time of emission. At a later point in time, the background concentration of CO_2_ is higher, and stopping or slowing down climate change becomes more costly [77]. The (expected) CO_2_ prices for 2010, and 2030 of the German Environment Agency are used [78]. These prices are also taken into account in Germany when planning infrastructure projects [79]. A constant growth rate of the CO_2_ price within the assessment period of this article from 2010 to 2030 is assumed and the prices are discounted with the consumer price index to the reference year 2019 [80]. Thus, the external costs of CO_2_ in the period under study increased from approximately 104 €/t CO_2_ in 2010 to ca. 189 €/t CO_2_ in 2030. The assumed costs for CO_2_ are also consistent with the average of recent studies [27,73]. The costs of the greenhouse gases N_2_O and CH_4_ were calculated as multiples of the CO_2_ prices, corresponding to their relative global warming potential (GWP_100_) in line with the fifth assessment report of the Intergovernmental Panel on Climate Change (IPCC) [81]. Numerous studies quantify the damage caused by GHGs in monetary terms. An EU study estimates between 60€_2016_ and 189€_2016_ per t CO_2_ in the period up to 2030, assuming a central value of 100€_2016_ per t CO_2_ [27]. A different study suggests a cost rate of 195€_2020_ per t CO_2_ in 2020 and 215 €_2020_ per t CO_2_ in 2030 taking a time preference rate of 1% into account (77). The external cost rate selected represents, to the knowledge, a value found reasonable by most studies. The cost rate is, of course, subject to large uncertainties. No one can precisely quantify the future damage caused by climate change. In the context of a sensitivity analysis, a current certificate price for CO_2_ [82] of approximately 75 €_November2022_ per t CO_2_ at the European ETS could serve as a lower bound.

The basis for the evaluation of the operation of nuclear power plants is the project ExternE [83], which considers the situation for Germany. The study was updated in 1999 and also considered the situation in other European countries [84]. However, the evaluation approaches are different in detail, and hence, are not comparable. The approach which considers the externalities caused by the operation was most extensively analyzed for the German nuclear power plants. Therefore, the assessment of the operation in all countries relies on the values for Germany [84]. Based on the nuclear accidents at Chernobyl and Fukushima, Rabl calculated the external cost rate for a nuclear accident per unit of energy [85], which is adopted for the Matrix C^k^. Furthermore, the external cost rates of the final disposal of fuel rods are taken from Rabl’s study [85]. All three external cost rates for nuclear energy were adjusted for the difference in purchasing power using the consumer price index.

The matrix C^k^ shown in Eq (3) has the same dimension for all countries in the analysis, as the 19 environmental externalities are documented in the rows and the 21 years of assessment in the columns.


$$\begin{array}{c} C^{k}=\left(\begin{array}{ccc} c_{11}^{k} & \cdots & c_{1|L|}^{k}\\ \vdots & \ddots & \vdots \\ c_{|I|1}^{k} & \cdots & c_{\left|I||L\right|}^{k} \end{array}\right)=(c_{il}^{k})\\ C^{k}\in R^{\left|I|\times |L\right|},i\in I=\{Externalities\},l\in L=\{Years\}=\{2010,\ldots ,2030\},k\in K=\{EU\;countries\},|I|=19,|L|=21 \end{array}$$ (3)

This article uses data on the population [86] and on the electricity demand [87] of European countries in the conclusion chapter to estimate the external costs of electricity production per inhabitant. Due to the Brexit, the energy demand of the UK is only available until October 2020 in these sources. Therefore, the energy demand for the UK was obtained from a national database [88].

### Estimating the external costs

This article is based on data on electricity mixes from 2010 to 2030 for 27 different countries in the European Union and emission data of the underlying conversion technologies for all countries from the life cycle assessment (LCA) database ProBas+ [63]. Furthermore, this article uses data on the external cost rates of 19 environmental externalities, mainly obtained from the NEEDS database [69].

After compiling the data, an individual Technology Matrix A^k^ for each country k has been created in the first step, which contains all conversion technologies used and the respective individual environmental externalities per kWh. These externalities can be divided into 3 globally impacting externalities and 16 locally impacting externalities. In the second step, the Electricity Mix Matrix B^k^, which contains the share of all technologies in the 21 observed years, and the External Costs Rates Matrix C^k^, which contains the cost rates of all environmental externalities in every year, has been calculated in the third step.

By firstly multiplying matrix A^k^ by matrix B^k^ and then multiplying the result element-wise by matrix C^k^, the average external costs of the electricity mix per kWh in each year for each country has been calculated in step four. The following Eq (4) describes the method:

$${ExtCosts}^{k}={(A}^{k}∙B^{k})\circ C^{k},k\in K=\{EU\;countries\},|K|=27.$$ (4)

Additionally, the external costs are divided into two categories. The external costs of all observed GHGs (CO_2_, CH_4_, and N_2_O) are classified as "global external costs" and all other external costs as "local external costs" in accordance with other studies [39,89]. One has to be aware that some externalities, such as a nuclear accident, might also have severe consequences for a broader geographic region. However, the impacts highly depend on the location of the emission and–unlike in the case of GHGs–are not uniform throughout the world. It should also be noted that due to the upstream chain of energy carriers, not all local impacts occur in the country of final electricity production. For example, the extraction of fossil fuels generates negative externalities [90]. These externalities are attributed to the country of final electricity production. The procedure also allows to determine the global and local external costs of each country for each year.

**Fig 1:**
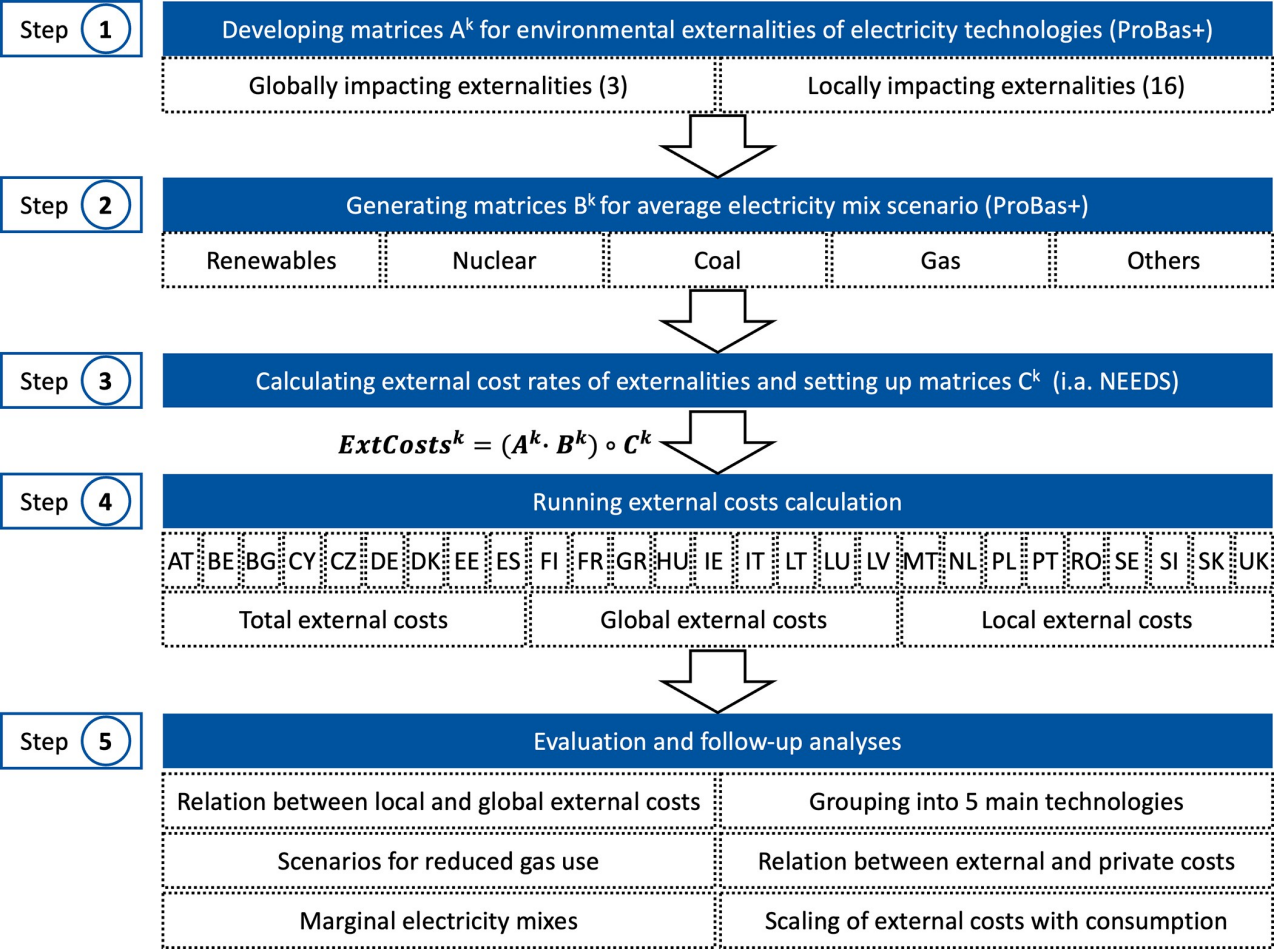
Research flow chart.

Based on the results of the calculation of the external costs, the results are finally evaluated in step five and follow-up analyses are carried out. Further analyses include the consideration of the correlation between local and global external costs, the grouping of the technologies into 5 main technologies, the analysis of scenarios for the reduced use of gas for electricity generation, the investigation of the correlation between the external and private costs of electricity, the consideration of marginal electricity mixes and the scaling of the external costs per unit of electricity. Fig 1 provides a research flow chart to visualize the overall process described in this chapter.

## Results

### The external costs of electricity in Europe

**Fig 2:**
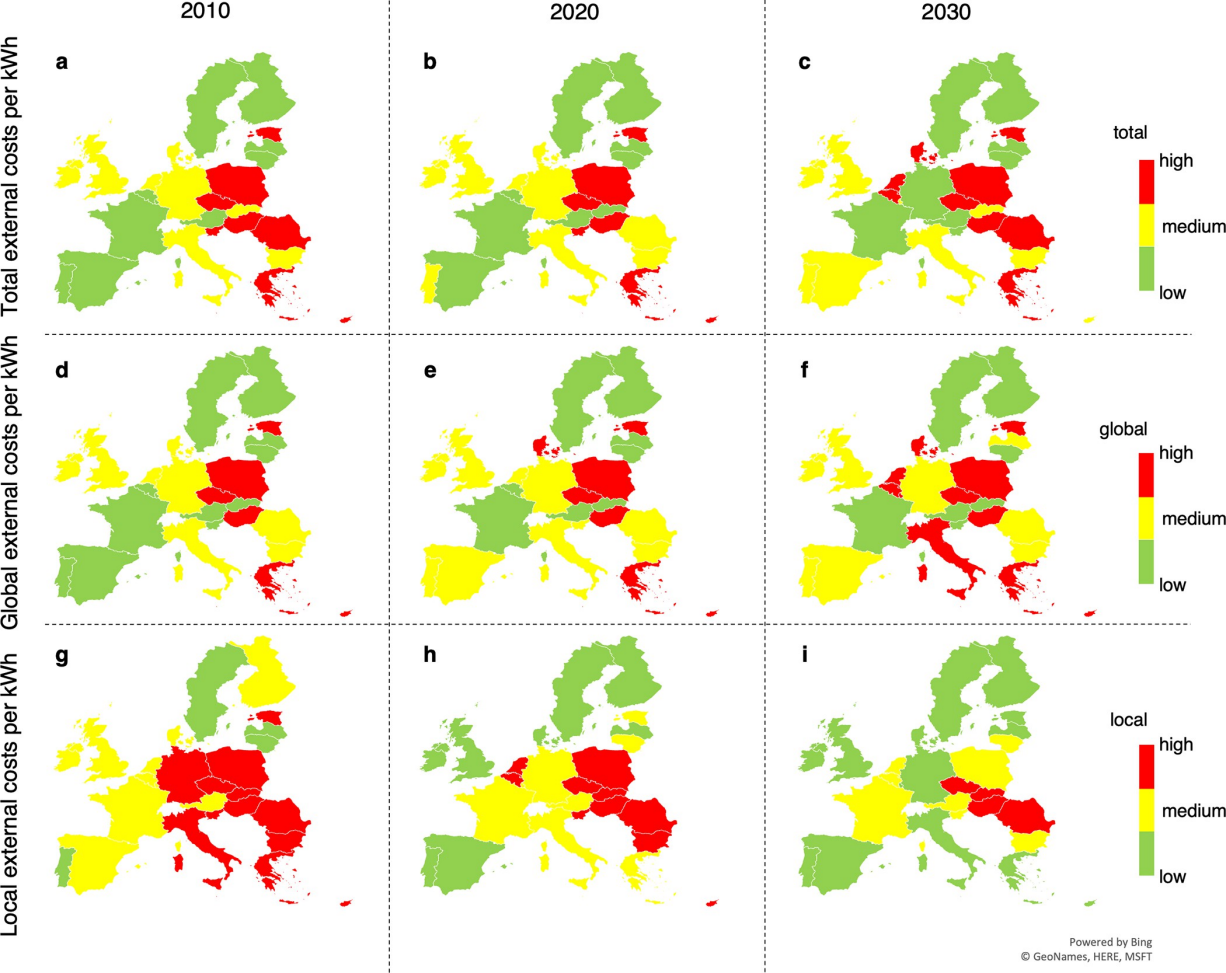
Map-based (relative) classification of total (a, b, c), global (d, e, f) and local (g, h, i) external costs of electricity in Europe in the years of 2010 (a, d, g), 2020 (b, e, h) and 2030 (c, f, i). The first row includes total external costs, the second row provides global external costs and the third row reports local external costs. For each of the perspectives, the figure presents the results for the years 2010, 2020, and 2030 and color them according to whether a country belongs to the third with the lowest costs (green), the third with medium costs (yellow) or the third with the highest costs (red).

In order to illustrate the relevant developments for Europe in the most effective way, the article focus the presentation on the years 2010, 2020, and 2030. Fig 2 provides a brief overview of the results for each country relative to the other countries.

A green classification is achieved with values of 2–5.8 (total), 1.1–4.9 (global) or 0.2–0.9 (local) cents per kWh. 6.2–9.3 (total), 5.1–7.3 (global) and 0.9–1.6 (local) cents per kWh are classified as yellow and values of 9.4–22.3 (total), 7.3–15.3 (global) and 1.6–12.1 (local) cents per kWh as red.

Overall, there are large disparities in Europe. External costs tend to be low in Scandinavia and comparatively high in Eastern Europe. Although the changes over time are not drastic, the trend is moving towards a reduction in local costs, whereas total and global costs are increasing. This trend is the most pronounced for those countries (Estonia, Germany, and Greece) where local external costs are moving from the worst third (red) to the best third (green). For global and total external costs, the trend is particularly pronounced for Belgium and for global external costs also for Luxembourg. While total external costs increase slightly, Cyprus, Germany, and especially Slovenia improve. For global external costs, no country is expected to improve its classification against the trend; for local external costs, only Lithuania upgrades its classification.

**Fig 3:**
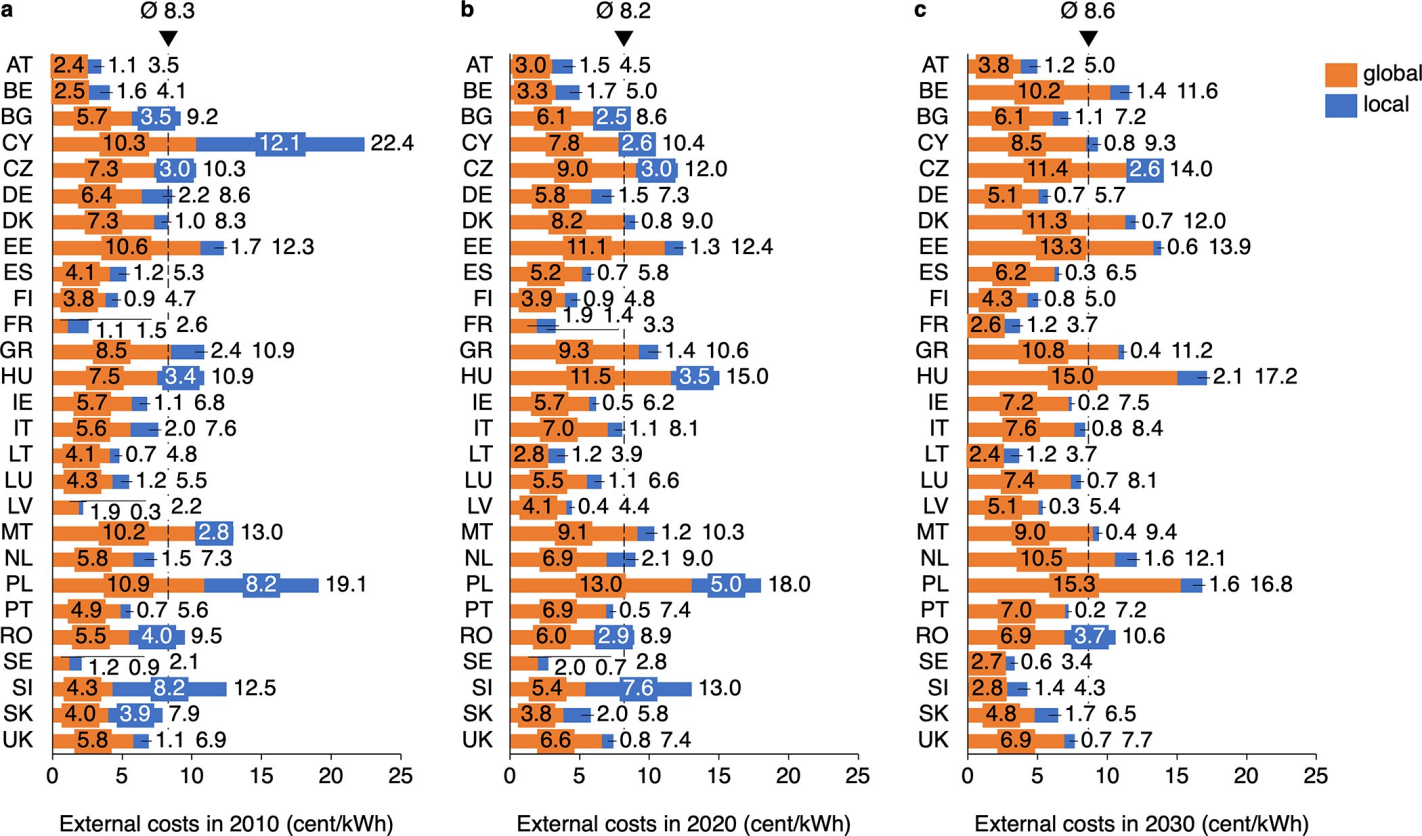
External costs of electricity in Europe in the years 2010 (a), 2020 (b) and 2030 (c) (absolute). The European country names in this and further figures are abbreviated as follows: Austria (AT), Belgium (BE), Bulgaria (BG), Cyprus (CY), Czechia (CZ), Germany (DE), Denmark (DK), Estonia (EE), Spain (ES), Finland (FI), France (FR), Greece (GR), Hungary (HU), Ireland (IE), Italy (IT), Lithuania (LT), Luxembourg (LU), Latvia (LV), Malta (MT), Netherlands (NL), Poland (PL), Portugal (PT), Romania (RO), Sweden (SE), Slovenia (SI), Slovakia (SK), United Kingdom (UK).

The stacked bar charts in Fig 3 present the external costs per kWh in 2010, 2020, and 2030, subdivided into global and local external costs. In contrast to Fig 2, absolute costs per kWh for the individual countries are reported.

In total, the costs rise by 0.4 cents per kWh. While local external costs fall from 2.7 to 1.1 cents, global costs increase from 5.6 to 7.6 cents on average, especially due to the progression of the CO_2_ price. Thus, the share of global external costs in the total external costs rises from about 70 to 86%.

Poland and Hungary have particularly high external costs over the entire period, while Cyprus can significantly reduce its high external costs from 2010 to 2030. Many countries have only minor differences in external costs over time. The share of local and global external costs in total external costs varies between countries and years. Amounts and shares of the costs depend on the country-specific external cost rates and individual generation technologies with, for example, different filter systems. France, for instance, which relies primarily on nuclear energy, has low external costs overall, but the proportion of local external costs is high.

**Fig 4:**
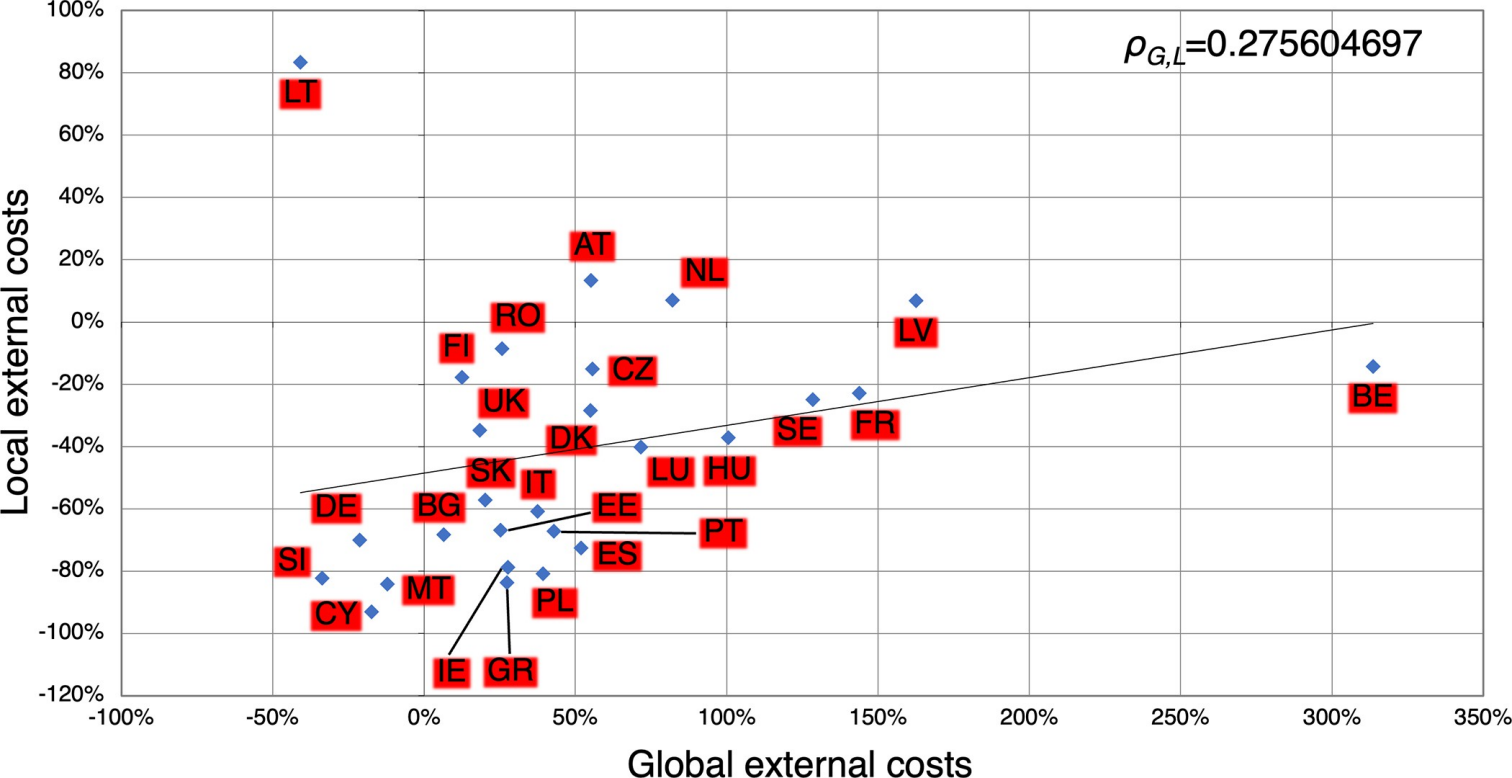
Change in local and global external costs from 2010 to 2030 in percentage points.

When comparing the long-term development of global and local external costs as depicted in Fig 4, it is questionable whether there will be a trade-off between the developments of global and local external costs or whether both will develop in the same direction. The trendline shows a weak positive correlation. The local and global externalities of countries per unit of electricity consequently change in the same direction over the analysis period, in line with other studies [91]. The coefficient of the Pearson correlation coefficient r_G,L_ is 0.276 but it is not significant (ρ_G,L_ = 0.164). The exceptional positions of Lithuania (strongly increasing local external costs and decreasing global external costs) and Belgium (sharply increasing global external costs) are striking and will be clarified in the chapter below. Omitting these outliers (or at least Lithuania) leads to a significant relation (α = 0.01).

### Assessment of electricity strategies

For the analysis of the results, the large variety of country-specific technologies have been grouped into five "basic” technologies: first "Renewable", which includes all forms of renewable energy, such as wind, water, photovoltaic, and biomass; second "Coal", which includes hard coal and lignite power stations; third "Nuclear", which includes all nuclear power stations; fourth "Gas", which includes all forms of electricity from natural gas; and finally, "Other", which includes all other electricity technologies, such as burning of oil or waste.

The grouping of the technologies allows to discuss different country strategies. Minimization strategies aim to minimize environmental risks or emissions and are therefore particularly relevant for nuclear energy and for CO_2_-intensive power generation from coal power plants. Maximization strategies concern the increase of energy from renewable sources [92,93], the switch to less CO_2_-intensive fossil energy from natural gas [94], or the increased use of nuclear energy [95,96]. Nuclear power is relatively low in pollutants but does carry risks. Different perceptions of these risks are the reason why some countries are investing in nuclear energy whereas others are phasing it out.

**Fig 5:**
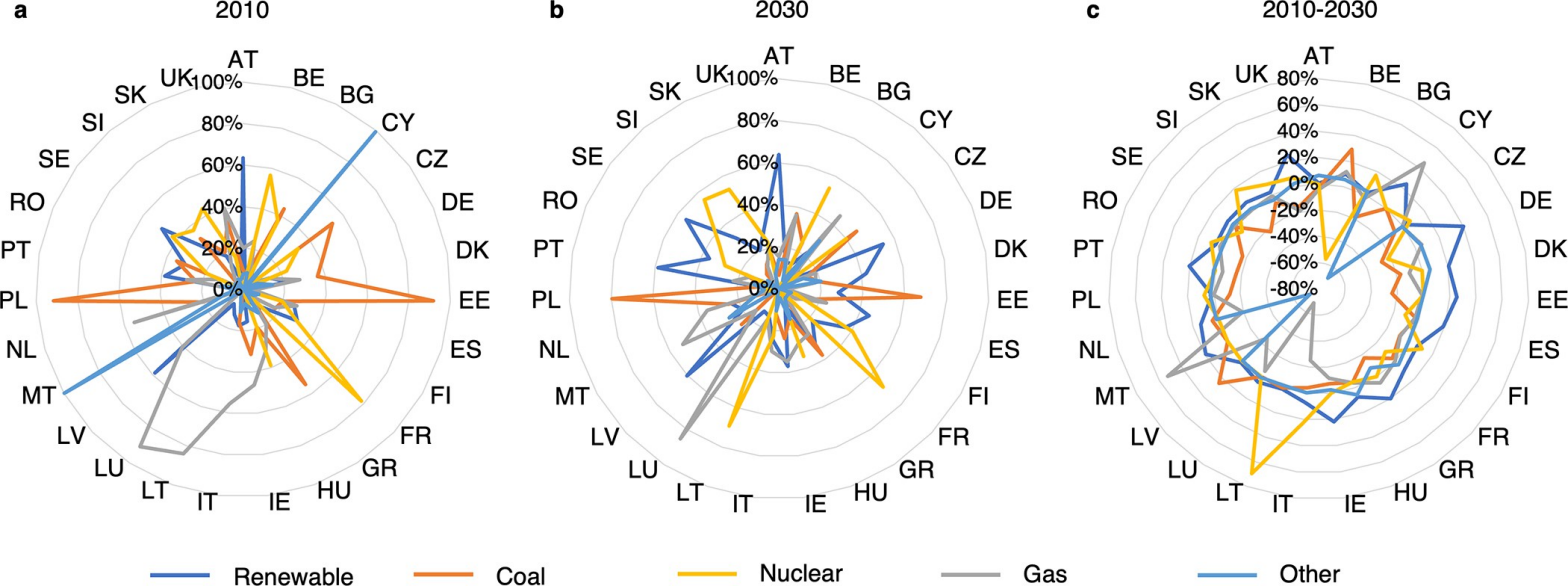
Electricity mix for the European countries in 2010 (a) and 2030 (b) and development of the electricity mix from 2010 to 2030 (c) in percentage points.

Fig 5 shows the electricity mix for each country for the years 2010 and 2030 and the development for the period 2010–2030. The high shares of nuclear energy use in France and of coal in Poland and Estonia are striking. Countries such as Austria, Latvia, and Sweden already had a large share of electricity from renewable sources in 2010, which is primarily due to their favorable geography and the resulting possibility of using hydropower. In 2010, the value for "Other" in Cyprus and Malta is very high at almost 100% and can be attributed to oil combustion. It is no coincidence that both countries are relatively remote island states.

There are notable differences between the energy mix in 2010 and that in 2030. The average share of renewable energies in the countries increases over this period to 30.3%, which corresponds to an increase of 12.0 percentage points. There is no decrease in this share in any country. Coal and gas are used 6.6% and 3.4% less, respectively. The share of nuclear power is increasing in 10 countries whereas it is decreasing in 7 countries, which will lead to an overall increase of 2.4%. Driven by Cyprus and Malta, the share of "Other" declines by 4.3%.

**Fig 6:**
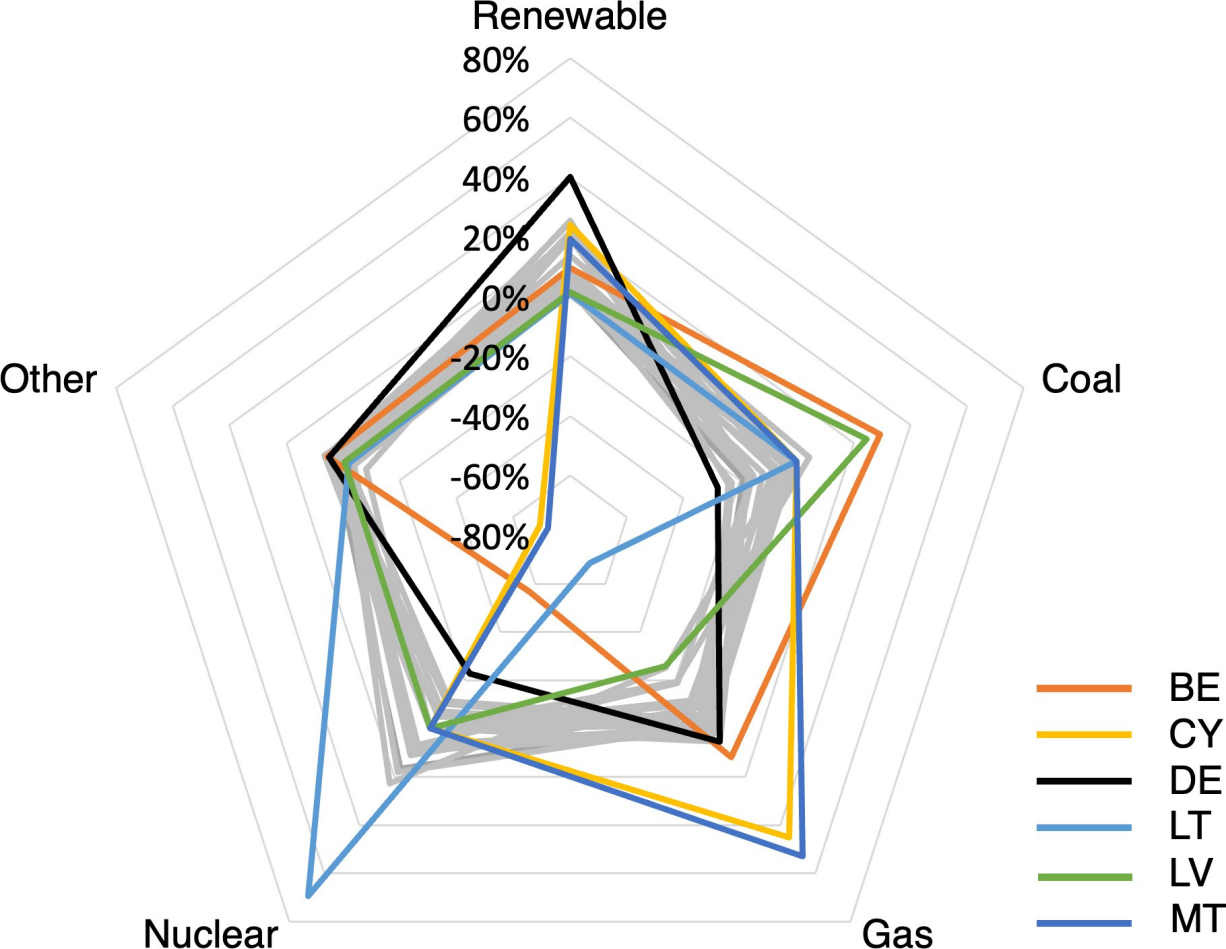
Development of electricity mix shares from 2010 to 2030 in percentage points.

To identify outliers in the strategies, a radar chart for the five basic technologies has been created, which is depicted in Fig 6. The chart presents all 27 countries according to their development of the electricity mix. Despite large differences in their respective energy production, most countries have relatively similar developments. For reasons of clarity, these countries are all colored gray and form a "belt". Six countries in particular stand out and warrant a more detailed discussion. These are: Belgium, Cyprus, Germany, Lithuania, Latvia, and Malta.

Belgium is one of the countries that had a relatively high share of nuclear energy of over 50% in 2010, but it will phase out this technology within the assessment period and will shut down its two power plants in Tihange and Doel [97]. Besides renewables and gas, Belgium is replacing most of its nuclear power capacity with coal-fired power plants. This is why the global external costs in Belgium are rising by far the most among all the countries. They even triple from 2010 to 2030.

Initially, Latvia primarily used natural gas and hydropower from the Daugava River, with both technologies being related to relatively low external costs. However, gas is being partly replaced by coal, so that external costs will increase slightly. Lithuania is reducing the share of natural gas even more significantly, with an initial share of almost 85%. Instead, plans involve building a nuclear power plant in Visaginas, but as the construction is controversial, the plans are currently on hold [98]. Nuclear power plants emit hardly any GHGs and thus have low global external costs. However, due to the externalities associated with radioactivity (radioactive radiation during regular operation, risk of accidents and final disposal of fuel rods), they have similarly high local external costs to conventional power plants. Due to the development and the higher local external costs of nuclear energy compared to gas turbines, the local external costs in Lithuania are increasing more than in any other country. However, the global external costs are decreasing as a result, so that in total the total external costs are reduced. These developments originate from tensions in these two Baltic states’ relations with Russia, which has cheaply supplied them with natural gas in the past. Hence, self-sufficiency efforts are playing a major role, and liquefied natural gas is increasingly being used.

Cyprus and Malta are largely replacing their oil-fired power plants with gas turbines. This switch is possible mainly due to new natural gas pipelines and discoveries of new gas reserves in the Levantine Sea [99,100]. By replacing the old power plants with modern gas turbines, local external costs are significantly reduced. Global external costs will also be lowered.

Germany is taking on a unique role [101]. Initially in 2010, the electricity mix consisted to a large extent of electricity from lignite, hard coal, and uranium. On the one hand, Germany, like Belgium, is opting out of nuclear energy, while at the same time reducing more than any other country its share of electricity generated from coal. It is largely replacing the lost capacities with energy from renewable sources. The share of renewable energies will increase by about 40%, which is also more than any other EU country. This enables Germany to reduce its global as well as local external costs. However, it is noteworthy that even Germany is likely to miss its GHG emissions targets by 2030, which are codified in the Paris Agreement and follow-up agreements [102].

Apart from the six countries discussed, the strategies of the other 21 countries are relatively similar: the use of coal and also gas is decreasing, compensated by an increasing share of energy from renewable sources. However, while countries "improve" their electricity mix and reduce emissions per kWh on average, only eight countries (Belgium, Cyprus, Germany, Lithuania, Malta, Poland, Slovenia, and Slovakia) can reduce their total external costs. Most of these countries had relatively high external cost levels in 2010, from which it is easier to improve. Four of these eight countries (Cyprus, Poland, Malta, and Slovenia) had the highest external costs of 2010.

Overall, most of the 27 EU countries have implemented a moderate change to their electricity mix. However, as greenhouse gases released in the future will cause increased external costs, it will hardly be possible to reduce external costs per kWh. Small changes to the energy mix will not be sufficient to counterbalance the expected increase of actual external costs, in particular for GHG emissions. An example of this is Denmark, which is increasing its share of renewable energies by 20% between 2010 and 2030 (primarily wind energy), but whose external costs are still rising by 3.7 cents per kWh. A total decrease in CO_2_ emissions of 16.3% is not sufficient to counterbalance the increase in the cost rate for CO_2_ of ca. 54% over the period. Hence, accelerating the transition of the energy systems in Europe will remain a politically pressing issue.

## Discussion

Electricity production in Europe is constantly undergoing transformations. In the past year, the dependence on natural gas from Russia became clear. Many countries, such as Germany, are trying to avoid natural gas as an electricity source [103]. In all the scenarios, the electricity mixes for the target year 2030 have therefore been adjusted so that all countries use 50% or 100% less natural gas. The gap was filled by all other technologies in proportion to their share in the national electricity mix. In terms of external costs, the differences regarding all 27 countries are rather small. On average, total external costs increase by 0.02% for a 50% reduction in natural gas use. Global external costs decrease on average by 1.57%, while the local external costs increase by 10.75%. This can be explained by the fact that the external costs of natural gas-fired technologies are in the middle-range of all technologies. Depending on the replacement technology, the impact varies from country to country. In Hungary, Malta and the Netherlands, the total external costs would increase by around 9–10%, as natural gas would be replaced to a significant extent by coal or oil. In Lithuania and Luxembourg, total external costs could decrease by around 11% as more environmentally friendly forms of energy would be used. Due to the composition of the electricity mixes in the different countires, the variation for a 100% reduction in natural gas consumption would be twice as high as for a 50% reduction.

While external costs reflect the unaccounted societal costs of electricity, there are also real costs for electricity. These costs are also referred to as private costs [104]. For policy recommendations, it would be of interest to know the relationship between the two. Different national taxation systems need to be taken into account in this regard. Taxes on electricity in the countries under study range from 4.8% in Malta to 68% in Denmark [105]. Therefore, electricity prices, excluding taxes and levies, are considered in this analysis [106]. For 2010 and 2020, these average prices per country are compared with the external cost results. However, there are no significant for the correlations between private costs and total, global, or local external costs. While the Pearson correlation between private and total external costs is slightly but not significantly positive in 2010 (r = 0.079, ρ = 0.695), this tendency is directly reversed in 2020 (r = 0.341, ρ = 0.082). The correlation in 2020 is also only significant at the 10% level. This trend would not be desirable from an incentive point of view, as countries with higher external costs would tend to have lower private electricity costs. As a result, taxes and levies would play an important role in achieving a desirable development. The exact drivers of this effect and their future development should be further investigated.

This article considers projected average annual electricity mixes per year in 27 EU countries. These electricity mixes are suitable for calculating the concrete damages of electricity in the respective year. Marginal electricity mixes are mostly used to estimate long-term technology impacts, which result, e.g., from capacity reductions and extensions of technologies. Based on the 2016 EU Reference Scenario [107], Vandepaer et al. determined marginal electricity mixes for the period from 2030 to 2040 [108], which are also applied in academia [46,109]. These mixes assess the changes in the energy mix due to a one-unit change in demand. Due to the fact that renewable energy capacity, in particular, is increasing in all countries, the marginal energy mixes, which are supposed to estimate the energy mix for 2040, have a share of renewable energy of at least 60% in all EU countries, of which mainly photovoltaics is the most important. Natural gas and, in some countries, nuclear power also play an important role. Coal is not part of the marginal electricity mix in any country, and the combustion of oil contributes less than 1.2% to the mix in each country. As a result, external costs would be significantly lower according to this electricity mix, which is intended to assess long-term developments. Assuming the 2030 external cost rates for comparability and applying this article’s technology data to the marginal electricity mixes, the external costs of Poland and Hungary, which have the highest external costs in 2030, would be reduced by 86% and 81%, respectively. In Sweden, which has the lowest external costs in 2030, the reduction potential would be 46%.

## Conclusion

The energy systems in European countries are undergoing substantial transformation processes. In particular, the EU’s climate targets aim at a more sustainable energy supply. Depending on the country involved, two low-carbon technologies–renewable energies and nuclear energy–are being pursued as options. This study shows that renewable energies are preferable, as they lead to the overall lowest external costs per kWh. Local external costs of a nuclear power plant are of a similar dimension to those of a fossil power plant. Nonetheless, nuclear energy could serve as a bridging technology. Switching to lower-carbon fossil fuels, such as gas, can also achieve significant reductions in external costs in the short term and therefore promote the sustainability of the energy sector.

The results support the EU’s ranking for nuclear and gas power [3,4], even though their external costs are higher than for renewable energy. However, the increased use of natural gas results in dependencies on countries with gas production and existing pipelines in the short term [110,111], which has become even more apparent after the Russian invasion of Ukraine. The phase-out of coal leads to a significant reduction in external costs. The benefit might be so substantial that reducing local external costs overcompensates the negative economic effects [112]. The reduction in global external costs, which only indirectly affects a country due to the worldwide consequences, would be a bonus benefit.

Each country has a different situation, so it is essential to analyze the exact circumstances, to overcome path dependencies and to accept the need for and to make appropriate changes. However, some of the underlying facts are uncertain and depend not only on political conditions and goals but also on external factors, such as technological developments, social beliefs, or the prices of resources [113]. Therefore, it is necessary to regularly reflect on the transition process and to adjust the strategy accordingly. At this point, it is important to emphasize the relevance of accurate and continuously updated electricity mixes. There are numerous, but no other electricity mix forecasts apart from the ProBas+ data used, which are based on a detailed technology basis.

External costs combine a variety of environmental externalities into a monetary measure. The results can be unterstood and interpreted by the general public [114] and raises awareness of the need to reduce individual ecological footprints. It would also make it possible to compare the environmental performance of countries and economies as a whole and to assess the effectiveness of policies in terms of their potential to reduce external costs. Similarly, taxes and subsidies can be assessed in terms of their appropriateness and level for improving environmental sustainability as measured by their potential to reduce external costs.

The method presented, which is based on country-specific emissions and individual external cost rates, is thus a suitable instrument for evaluating the sustainability of energy technologies on a country-specific basis. When newer data on future electricity mixes and their emissions become available, the model can prospectively be updated. The open accessibility of data is limited, especially in energy research [115]. Improvement would be desirable here.

One limitation arises from the assumption of average electricity mixes. In order to evaluate individual electricity consumption from the perspective of external costs, a much more granular approach would be required since there are considerable temporal and local fluctuations in the electricity mix in countries within a year. Inaccuracies also arise from the fact that only cradle-to-gate emissions data are available, as the editors of the ProBas+ database have added a 5% surcharge to emissions to extend the dimension to cradle-to-grave [63], but at least country-specific individual values for grid losses would be desirable here. This would allow for an individual evaluation of entities, whereas the focus of this study is on an average approach, which allows for an estimation of the total external costs due of electricity generation in countries.

For the energy development scenarios, the work is limited to the assumptions of the ProBas+ database [63]. The development of electricity mixes in Europe is very dynamically. Energy scenarios, as well as the electricity mixes used, are updated relatively infrequently, but this would increase the predictive power of future research. Databases should also, where possible, include different scenarios of electricity mixes and resulting emissions to show different development paths, as many parameters of future development are uncertain. In this sense, a uniform reporting requirement would be desirable to make electricity emissions more transparent to researchers and the public. In addition to the average electricity mix and its emissions, the marginal mix and its emissions would also be of interest. This would also allow the future potential of electricity to be quantified more accurately. In addition, a comparison of the database used, ProBas+ [63] with other emission databases, such as Ecoinvent [116], could provide additional information.

External cost rates are subject to considerable uncertainty. Particularly for air pollutants, cost rates can vary widely between locations [117]. Hence, country-specific external cost rates has been and should be used for each pollutant [69]. For PM, more specific cost rates already exist [73], but there is still a need for extensive research on fine-grained external cost rates. It is important to emphasize that external cost rates differ between countries for most emissions. In this study, all emissions resulting from electricity generation in a country have been attributed to that country. For many energy sources, parts of the value chain take place in other countries. Therefore, data on the specific allocation would be desirable for a more accurate calculation of the external costs.

By combining the research results with national electricity consumption [87], the entire external costs of using electricity in the EU in 2020 amount to approximately 197 billion €. This corresponds to average external costs of 385 € per capita. These external costs vary significantly between 156 € per capita in Latvia and 811 € per capita in Estonia, due to the highly divergent external costs of the respective electricity mix and the different levels of energy consumption per capita.

These results do not only call for radical changes but also for country-specific measures to reduce the external costs per kWh. Nevertheless, saving electricity is as important as generating it as sustainably as possible, and there is considerable potential for savings within Europe [118,119]. In this vein, the Smart Energy Systems concept offers the possibility to adjust the demand for electricity to the availability of renewable electricity and to couple different sectors in order to reduce external costs in the future [120,121].

In summary, this article describes the average external costs for one unit of electricity in different European countries. Through this approach, benchmarking between different countries can be easily carried out, and recommendations for action can be derived. However, this article does not address the difference between relative and absolute amounts of externalities caused per country. Thus, any changes to the energy system of a larger country have, of course, a more significant impact. Furthermore, the risk of burden-shifting to outside Europe and temporal profiles of renewable electricity generation cannot considered by analyzing an average unit of electricity.
